# The role of area level social deprivation on childhood and adolescent consultation rate in primary care: a population based, cohort study

**DOI:** 10.1186/s12875-022-01873-x

**Published:** 2022-10-28

**Authors:** M. S. Fonderson, P. J. E. Bindels, A. M. Bohnen, E. I. T. de Schepper

**Affiliations:** Department of General Practice, Erasmus Medical Center, PO Box 2040, 3000 CA Rotterdam, the Netherlands

**Keywords:** Children, Adolescent, General practice, Consultation rates, ICPC-1, Area level social deprivation

## Abstract

**Background:**

Studies show that children and adolescents in the most socially deprived areas (SDA) consult their general practitioner (GP) more often than those in the least socially deprived areas (Non-SDA). Given that GPs see a wide range of diseases, it is important to know which clinical diagnoses are shaped by socioeconomic factors. The primary objective was to determine the association between area level social deprivation and consultation rates in a pediatric population. The secondary objective was to explore this association across a wide range of clinical diagnoses.

**Methods:**

A cohort study using the Rijnmond Primary Care Database (RPCD) was conducted. Between 2013 and 2020, a total of 69,861 patients aged 0 to 17 years registered with a GP were analysed. A consultation was defined as patient contact and entry of a diagnosis using the International Classification of Primary Care (ICPC-1) code. Associations between consultation rates, ICPC-1 codes and area level social deprivation were explored using a Poisson regression model. The incidence risk ratio (IRR) and 95% confidence interval (CI) were reported.

**Results:**

Over the 7-year study period the consultation rate of the study population was 3.8 per person-years. The top 5 reasons for children and adolescents to consult their GP was related to skin, respiratory, general unspecified, musculoskeletal and digestive symptoms or diagnoses. Consultation rate was higher in SDA group compared to Non-SDA group (IRR 1.20, 95% CI 1.19–1.20). Consultation rate for ICPC-1 code related to pregnancy and family planning was significantly lower in SDA group compared to Non-SDA group. Upon further exploration of this code, SDA group were less likely to consult for oral contraception and more likely to contact a GP for induced termination of pregnancy compared to Non-SDA group (IRR 0.36; 95% CI 0.33–0.44 and IRR 2.94; 95% CI 1.58–5.46 respectively).

**Conclusions:**

Overall, SDA group had higher GP consultation rates for the majority of clinical diagnoses except for pregnancy and family planning. In this latter category, adolescent females in SDA consulted less frequently for oral contraception. This study illustrates the need to understand the underlying health seeking behaviors of children and adolescents at different development phases of their lives.

**Supplementary Information:**

The online version contains supplementary material available at 10.1186/s12875-022-01873-x.

## Background

With a projected two-thirds of the world population living in metropolitan areas by 2050, changes in neighborhood infrastructure and ethnic heterogeneity [1] will likely affect health risks and outcomes [2]. Access to primary care services is under pressure [3]. Particularly in metropolitan cities with higher population density, factors such as age and socioeconomic factors play a role in consultation with a general practitioner (GP) [4–6]. In the Netherlands, 77% of children consult their GP at least once a year for different reasons ranging from acute symptoms to preventive counseling [4].

The role of area level social deprivation and GP consultation rates in children has been studied. There is evidence that poorer health, less access to preventive care and increased staff contributes to increased consultation rates in children from most deprived areas compared to children who live in least deprived areas [3, 7]. In contrast, a recent study found that children from most deprived areas had fewer consultations compared to children in least deprived areas, which was in part due to higher visits to unscheduled (emergency) care [8]. Findings on clinical conditions related to high consultation rates are limited and if available, report on single diagnosis such as knee pain, obesity, allergic rhinitis and depressive symptoms in children or adolescents [9–12]. To address this knowledge gap, we explored trends in primary care visits among children within a metropolitan area. Given that Dutch GPs are allocated additional funding based on patients who live in deprived areas, a study on a range of clinical reasons for encounters in this setting was performed in order to make comparisons across different area level social deprivation groups. Particularly in the context of how clinical diagnoses are likely shaped by socioeconomic factors during critical phases of development from childhood to adolescence.

We therefore hypothesize that in a metropolitan city such as Rotterdam, children from most deprived areas consult their GPs more frequently than children from least deprived areas. The primary aim of this study was to describe and analyse the extent to which area level social deprivation contributed to health-seeking behavior of children and their parents or caregivers over time, both in terms of consultation rates and reasons for encounters. In the event that an observed trend was significantly different from our hypothesis, our secondary aim was to explore the clinical context of these findings.

## Methods

### Data sources

We conducted a population based cohort study using the Rijnmond Primary Care database (RPCD). The RPCD is a region-specific derivative of the Integrated Primary Care Information (IPCI) database, under the supervision of the Department of General Practice of the Erasmus Medical Center [13]. The IPCI database is a medical record database and details on this are published elsewhere [14, 15]. The medical records of patients in the RPCD are pseudonymised and contain information on both patient demographics (date of birth, sex, area level social deprivation) and ICPC-1 codes for clinical characteristics (signs, symptoms, diagnoses, physical findings, laboratory test results, drug prescriptions, referral to specialists and hospitalization).

Data collection commenced in 2011 and over time a growing number of GPs contributed to over 200,000 medical records from patients who live in the greater area of Rotterdam located in the province of South-Holland, the Netherlands. Rotterdam has 9 residential areas, 22 districts and 99 neighborhoods (supplementary 2). Each neighborhood has a unique postcode made up of 4 numbers followed by 2 letters. Some postcodes with identical 4-number combinations are shared by more than one neighborhood. There are nearly 330,000 households and approximately 30% of these households have children. This area consists of varying socio-demographic composition and is broadly representative of general practices nationwide in terms of size and patient access to a GP. The use of this database for the current study was approved by the Rijnmond Primary Care scientific and ethical advisory group.

### Study population

Our study consisted of children aged 0 to 17 years old registered with a GP practice between 01 January 2013 and 31 December 2019.

We included those who contributed at least 1 year of valid electronic database information. Follow-up ended on the date one of the following events occurred: the child transferred out of the practice, the GP practice stopped data collection, the child died, or data collection for this study was complete (31 December 2019). Data from the year 2020 are not reported due to the changes in utilisation of healthcare services as a result of the COVID-19 pandemic.

### Outcomes

The primary outcome of this study was consultation rate calculated as the total number of consultations divided by person-year. We calculated person-year of observation for each age and sex strata over the 7-year study period. Crude rates were calculated for each year of observation.

The secondary outcome of this study was consultation rates in the clinical context using ICPC-1 chapter codes. We calculated risk ratios in SDA and Non-SDA groups.

### Variables

#### Number of consultations

A consultation was defined as contact that took place either face-to-face, electronically (email or telephone) or home visit between a child and a GP or practice nurse. The RPCD stores unique contact identification (ID) numbers per child. Every entry entered by a GP as free-text in the SOEP (Subjective, Objective, Evaluation, Plan) electronic record field is stored as a unique contact ID. Patient-specific variables attached to each contact ID were extracted and included age, sex, area level social deprivation category, date of consultation and ICPC-1 code. A unique contact can have several ICPC-1 codes and is considered as one consultation. We were able to extract the number of consultations per child per year and subsequently categorized them into 5 different groups (0 consultations; 1–2 consultations; 3–5 consultations; 6–10 consultations; more than 10 consultations).

#### Age

We calculated the age of children in mid-July of that year during the study period 2013 and 2019. Children were subsequently categorized into three age groups (0–5 years; 6–11 years; 12–17 years). Age at consultation was derived from a child’s age that was linked to the date of consultation.

#### Sex

We extracted data on sex and recoded this into categorical variables; Male or Female.

### Area level social deprivation

Funding for GP care is based on a system whereby additional resources are made available for practices where residents live in areas with relatively high deprivation. These areas are described by the Dutch Healthcare Authority (NZA) [16] on the basis of a postcode list (postal code level 5 (4 numbers and one letter) and postal code level 6 (4 numbers and two letters)). The list is provided by the Central Bureau of Statistics (CBS) [17] to calculate the area level social deprivation (SDA) index. This index is the sum of standardized scores derived from four indicators: benefit recipients, residents with low income, surrounding address density and non-western foreigners. The formula used for social deprivation index has been described elsewhere [18]. In short, the social deprivation index uses standardized variables (variable value minus mean value, divided by standard deviation). Means and standard deviation are weighted by the number of residents per neighborhood. Due to the skewed distribution of variables used, a transformation to the natural logarithm (ln) of the variables is performed first before they are weighed. The formula used to compose the 2012 SDA index is.

$$\left(\left(\mathit{\ln}\_ percentage\ of\ social\ benefit\ recipients/ non- students-2.7110\right)/0.42360\right)+\left(\left(\mathit{\ln}\_ percentage\ of\ low- income\ residents-3.6640\right)/0.21573\right)+\left(\left(\mathit{\ln}\_ surrounding\ address\ density-7.0546\right)/1.05123\right)+\Big(\left(\mathit{\ln}\_ percentage\ of\ non- western\ immigrants-1.8077\right)/1.17265.$$

$$\ast \mathit{\ln}: the\ natural\ logarithm.$$

$$\ast surrounding\ address\ density: the\ average\ number\ of\ addresses\ within\ a\ 1\; km\ radius.$$

In 2019, the population of the greater area of Rotterdam was 650,980 inhabitants of which 371,025 (57%) lived in Non-SDA and the remainder 279,955 (43%) lived in SDA. Of the 99 neighborhoods in Rotterdam, 38 of them were identified by the NZA as SDA and 61 as Non-SDA. The mean number of inhabitants in SDA and Non-SDA were 7367.2 and 6082.4 respectively (Additional file 1 Appendix 1).

The healthcare records of the RCPD documented area level social deprivation (SDA) in a binary (yes/no) format. This variable was re-coded into living in most socially deprived area (SDA group) and living in least socially deprived area (Non-SDA group). Based on this variable, it is not possible to identify the child nor the neighborhood he or she lives in.

### International classification of primary care (ICPC-1)

The International Classification of Primary Care (ICPC-1) is a recognized tool to encode primary care medical reason for encounters (RFEs). Classifying each consultation with an ICPC-1 code provides an indication of the prevalence of illness [19–22] and also serves as a proxy for the GPs clinical workload [6, 21, 23]. ICPC-1 is based on a bi-axial structure: 17 chapters categorized according to body systems with an alpha code on one axis and seven identical components with a two-digit numeric code on the second axis [24]. The seven components are further distinguished as symptoms (component 1); diagnostic, screening and preventive procedures (component 2); medication, treatment and procedures (component 3); test results (component 4); administration notes (component 5); referrals and other reasons for encounter (component 6); or diseases (component 7). Within component 7 are five subgroups (infectious diseases, neoplasms, injuries, congenital anomalies and other diseases). In this study we included diagnostic codes for components 1 and 7 which are independent of the body systems and can be used to code patient RFEs.

### Statistical analysis

Continuous data (age, number of consultations) were presented as mean (standard deviation, SD) if normally distributed or median (Interquartile range, IQR) if skewed. Categorical data (ICPC-1 codes, sex, age category, number of consultations category, social deprivation score) were presented as frequency (%). We used a Poisson regression analysis to model the incidence risk ratio (IRR) and the 95% confidence interval (95% CI) relative to children’s age, sex and area they lived in. No selection procedure was used since previous studies have shown these variables to be predictors of GP consultations [7, 23, 25, 26]. All analyses were performed in R studio, version 1.2.

## Results

The characteristics of 69,861 children registered with a GP throughout the entire study period 2013 to 2019 are shown in Table 1. Each year there was a steady increase of GP practices who provided data for the RPCD. Overall, females accounted for 49% of the patient population and there were slightly more children in the 0–5 years age category (35%) compared to those in age category 12–17 years (33%) and 6–11 years (32%). The median number of consultations in the study population was 2 (IQR: 0–5). Each year, approximately 25% of children did not consult their GP (0 consultations). One-third (33%) of children consulted their GP once or twice a year. Twenty-three percent of children had 3–5 consultations per year compared to 14% who had between 6 and 10 consultations per year with their GP. Just under 10% of children consulted their GP more than 10 times a year. Overall, 50,107 patients (71.2%) lived in the least deprived areas (Non-SDA) and 17,623 lived in the most deprived areas (SDA). A total of 2131 (3.05%) children had missing area level social deprivation data.

**Table 1:** Characteristics of children registered with a GP between 2013 and 2019

Characteristics	2013	2014	2015	2016	2017	2018	2019
**Registered children, N**	33,281	34,492	35,206	39,938	44,937	44,992	46,493
**Female, N (%)**	16,377 (49.2)	16,962 (49.2)	17,321 (49.2)	19,636 (49.2)	22,053 (49.1)	22,058 (49.0)	22,751 (48.9)
**Age category, N (%)**							
0–5 years	11,727 (35.2)	12,244 (35.5)	12,416 (35.3)	14,025 (35.1)	15,569 (34.6)	15,433 (34.3)	15,829 (34.0)
6–11 years	10,666 (32.0)	10,985 (31.8)	11,166 (31.7)	12,714 (31.8)	14,523 (32.3)	14,844 (33.0)	15,435 (33.2)
12–17 years	10,888 (32.7)	11,263 (32.7)	11,624 (33.0)	13,199 (33.0)	14,845 (33.0)	14,715 (32.7)	15,229 (32.8)
**Consultation category, N (%)**							
0	9135 (27.4)	8270 (24.0)	8319 (23.6)	9714 (24.3)	12,087 (26.9)	11,867 (26.4)	11,921 (25.6)
1–2	9948 (29.9)	10,197 (29.6)	10,468 (29.7)	11,742 (29.4)	13,245 (29.5)	12,661 (28.1)	13,486 29.0)
3–5	7751 (23.3)	8534 (24.7)	8724 (24.8)	9852 (24.7)	10,323 (23.0)	10,516 (23.4)	10,641 (22.9)
6–10	4638 (13.9)	5323 (15.4)	5359 (15.2)	5981 (15.0)	6495 (14.5)	6764 (15.0)	7094 (15.3)
> 10	1809 (5.4)	2168 (6.3)	2336 (6.6)	2649 (6.6)	2787 (6.2)	3184 (7.1)	3351 (7.2)

### Number of consultations

There was a total of 964,123 consultations recorded in 69,861 registered children (251,136 person-years) over a 7-year period, this resulted in an overall consultation rate of 3.8 per person-years (Table 2). Consultation rates rose from 3.7 per person-years in 2013 to 4.0 per person-years in 2019. Overall, consultation rates for females and males were 3.97 per person years and 3.71 per person-years respectively. In our study population, consultation rates varied by age category. The highest consultation rate occurred in the age category 0–5 years (4.75 per person-years) compared to a consultation of 3.23 per person-years in age category 6–11 years and 3.57 per person-years in age category 12–17 years.

**Table 2:** Characteristics of consultations (*N* = 964,123) recorded by GPs between 2013 and 2019

Characteristics	2013	2014	2015	2016	2017	2018	2019
**Total Consultations, N**	105,573	119,834	124,040	140,305	150,392	158,931	165,048
**Female, N (%)**	53,853 (51.0)	60,775 (50.7)	62,776 (50.6)	70,808 (50.5)	76,545 (50.9)	81,388 (51.2)	84,049 (50.9)
**Age category, N (%)**							
0–5 years	44,226 (41.9)	49,221 (41.7)	51,384 (41.4)	56,881 (40.5)	59,567 (39.6)	61,864 (38.9)	64,428 (39.0)
6–11 years	28,931 (27.4)	33,132 (27.6)	33,761 (27.2)	39,281 (28.0)	42,095 (28.0)	45,387 (28.6)	48,021 (29.0)
12–17 years	32,416 (30.7)	37,481 (31.2)	38,895 (31.4)	44,143 (31.5)	48,730 (32.4)	51,680 (32.5)	52,599 (31.9)
**Total person-years, N**	28,928.8	31,746.1	32,496.1	36,588.4	39,552.7	40,667.3	41,156.5
**Consultation rate per person-years**	3.65	3.77	3.82	3.83	3.80	3.91	4.01

### Risk factors related to consultations

Table 3 shows the risk factors related to consultations in the study population. After controlling for age and area level social deprivation, females consulted a GP more often compared to males (IRR 1.07, 95% CI 1.07–1.08). Children in age category 6–11 years and 12–17 years had fewer consultations with their GP (IRR 0.74, 95% CI 0.74–0.76 and IRR 0.84, 95% CI 0.83–0.84 respectively) compared to children in age category 0–5 years. During the 7-year period, children living in SDA consulted their GPs more often compared to those living in Non-SDA (IRR 1.20, 95% CI 1.19–1.20).

**Table 3:** Relative risk and 95% confidence interval (CI) of annual consultation rates in children between 2013 and 2019 by sex, age category, living in SDA

	2013 RR (95% CI)	2014 RR (95% CI)	2015 RR (95% CI)	2016 RR (95% CI)	2017 RR (95% CI)	2018 RR (95% CI)	2019 RR (95% CI)
**Intercept**	3.41 (3.37–3.45)	3.70 (3.66–3.74)	3.79 (3.75–3.83)	3.71 (3.67–3.74)	3.54 (3.50–3.58)	3.83 (3.78–3.86)	3.77 (3.74–3.81)
**Female sex**	1.07 (1.06–1.09)	1.06 (1.05–1.07)	1.06 (1.05–1.07)	1.06 (1.04–1.07)	1.08 (1.07–1.09)	1.09 (1.08–1.10)	1.08 (1.07–1.10)
**Age category 6–11 years** ^**a**^	0.72 (0.71–0.73)	0.75 (0.74–0.76)	0.73 (0.72–0.74)	0.76 (0.75–.077)	0.76 (0.75–0.77)	0.76 (0.75–0.77)	0.77 (0.76–0.77)
**Age category 12–17 years**^**a**^	0.79 (0.78–0.80)	0.83 (0.82–0.84)	0.81 (0.80–0.82)	0.83 (0.81–0.84)	0.86 (0.85–0.87)	0.87 (0.86–0.88)	0.85 (0.84–0.86)
**Living in SDA**^**b**^	1.26 (1.25–1.28)	1.23 (1.21–1.24)	1.25 (1.24–1.27)	1.27 (1.26–1.28)	1.20 (1.19–1.21)	1.09 (1.07–1.10)	1.16 (1.14–1.17)

*All coefficients were significant at *p*-values < 0.001

^a^Age category 0–5 years as reference

^b^Non-SDA as reference

### Reasons for encounters and role of area level social deprivation

**Fig. 1:**
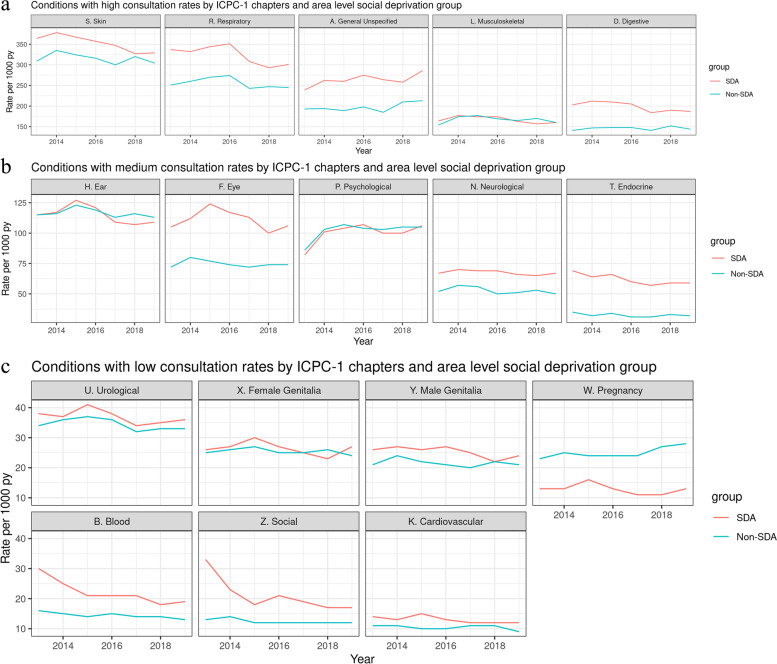
**a** Consultation rates by ICPC-1 chapters and area level social deprivation group: Conditions with consultation rates higher than 150 per 1000 person-years (py). **b** Consultation rates by ICPC-1 chapters and area level social deprivation group Conditions with consultation rates between 51 and 149 per 1000 person-years (py). **c** Consultation rates by ICPC-1 chapters and area level social deprivation group. Conditions with consultation rates lower than 50 per 1000 person-years (py)

During the 7-year study period, GPs recorded 992,482 ICPC-1 codes in consultations with children. Higher GP consultation rates (more than 150 per 1000 person-years) were found in ICPC-1 chapters related to skin, respiratory, general unspecified, musculoskeletal and digestive (Fig. 1a). Consultation rates lower than 50 per 1000 person-years were found in ICPC-1 chapters related to urological, female genitalia, male genitalia, blood, pregnancy, social problems and cardiovascular diseases (Fig. 1b). For ICPC-1 chapters related to ear, eye, psychological, neurological and endocrine, consultation rates were between 51 and 149 per 1000 person-years (Fig. 1c).

Annual trends of consultation rates per ICPC-1 chapter according to area level social deprivation group are found in Fig. 1 a, b and c. The figures show that in general children in SDA group consulted their GPs more frequently for 14 of the 17 ICPC-1 chapters. Consultation rates did not significantly differ between areal level social deprivation when children consulted for musculoskeletal or psychological reasons. Our results show that the ICPC-1 chapter relating to pregnancy and family planning was the only category in which consultation rates was significantly lower in SDA group compared to the Non-SDA group.

**Fig. 2:**
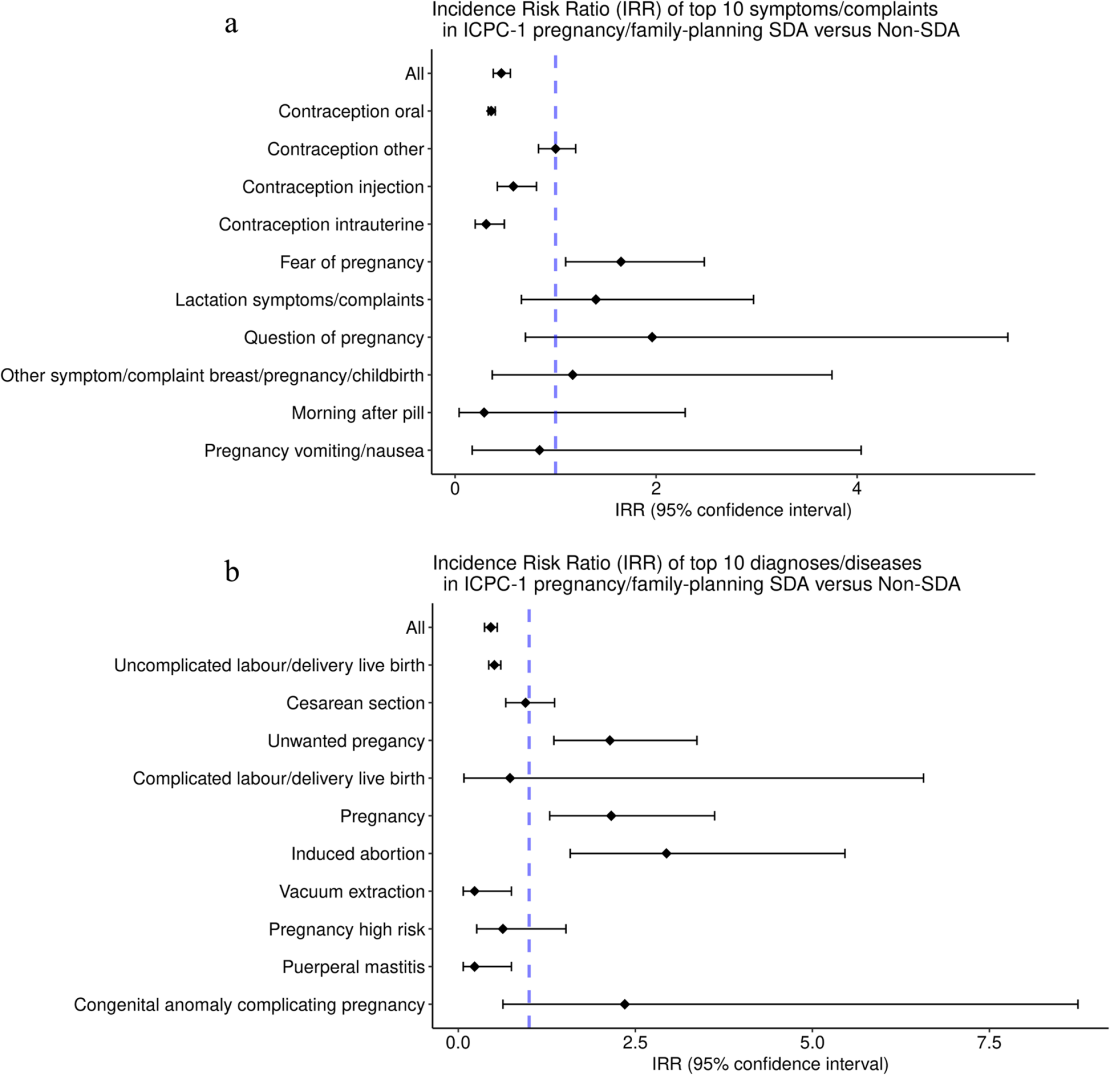
**a** Forest plot of incidence risk ratio (IRR) of top 10 symptoms and complaints in ICPC-1 chapter pregnancy & family planning. **b** Forest plot of incidence risk ratio (IRR) of top 10 diagnoses and diseases in ICPC-1 chapter pregnancy & family planning

Upon further exploration of this chapter, the following findings became apparent. In symptoms related to pregnancy, the most common reason for consultation with a GP was for contraceptives (Additional file 1 Appendix 2). SDA group consulted their GP less frequently for oral contraception (IRR 0.36; 95% CI 0.33–0.44), intrauterine device (IUD) contraception (IRR 0.31; 95% CI 0.20–0.49) and injection contraception (IRR 0.58; 95% CI 0.42–0.81) compared to Non-SDA group (Fig. 2a). In contrast, GPs recorded higher rate of consultations in SDA group for confirmed pregnancy (IRR 2.16; 95% CI 1.29–3.62) and unwanted pregnancy (IRR 2.14; 95% CI 1.35–3.37) compared to Non-SDA group (Fig. 2b).

In our study population, those in SDA group consulted their GPs three times more often for induced termination of pregnancy (IRR 2.94; 95% CI 1.58–5.46) compared to those in Non-SDA group (Fig. 2a and b).

Of all consultations related to pregnancy and family planning 11% were for unwanted pregnancies and/or induced termination of pregnancy. Consultation rates of unwanted pregnancies were higher in female adolescents in SDA group (0.85 per 1000 person-years) compared to those in Non-SDA group (0.34 per 1000 person-years) (Additional file 1 Appendix 2).

## Discussion

### Principle findings

This is a large-scale cohort study that describes the association between area level social deprivation and childhood consultation rates. Between 2013 and 2017, a rise in consultation rate was observed in children who attended general practice services. The trend found in this study is similar to national trends in consultation rates [27]. Overall consultation rate in children attending general practice services was 3.80 per person-years. Children from most socially deprived areas consulted a GP more frequently for the vast majority of clinical diagnosis compared to children from least socially deprived areas.

For the vast majority of the ICPC-1 chapters, children in SDA group had higher GP consultation rates compared to children in Non-SDA group. However, in the pregnancy/child bearing and family planning ICPC-1 chapter, adolescent females in SDA group were less likely to consult their GPs compared to adolescent females in Non-SDA group. Further analysis revealed that adolescent females from most deprived areas consult their GP less frequently for contraceptives (oral, injection or intrauterine) compared to adolescent females from Non-SDA group. Consequently, confirmed pregnancy, unwanted pregnancy and induced termination of pregnancy were more often observed in adolescent females in SDA group.

### Comparison with other studies

With regards to consultation rates in general practice, the findings of our study are largely consistent with the literature. A few studies in the Netherlands show that GPs record between 3 to 7 consultations per child per year [25, 27, 28]. This is similar to studies performed in other countries, where consultation rates in children range between 2 to 7 per year [3, 26, 29, 30]. With regards to the effect of socioeconomic factors, our observation that children from most deprived areas consult their GPs 20% more frequently than those from least deprived areas is also largely consistent with the literature. Bruijnzeels et al., [31] found that the odds of consultation rate was 24% higher in children from low/middle socioeconomic status.

(SES) compared to those from high SES. Elsewhere, Saxena et al., [3] showed that consultation rates were 18% higher in children from social classes IV-V (lowest social class) than in children from social classes I-II (highest social class). McLeod [32] observed children aged younger than 6 years and living in the most deprived areas consulted their GP 16% more frequently than those in the same age category living in least deprived areas. In their multivariate analysis, Mukhtar et al., [7] found an 18% higher consultation rate in the group of patients with an IMD (index of multiple deprivation) score in the 5th quintile (most deprived).

In contrast to these findings, a recent study in England showed that children from most deprived areas accounted for 4.8% fewer GP consultations compared to children from least deprived areas [8]. The authors argued that this decreased trend was due in part to a shift towards emergency department visits by children from most deprived areas. Given that GPs in deprived area have a 15% increased workload [33], a shift to unscheduled care (i.e., out-of-hours or emergency care services) is a likely consequence. This was observed in a study were children aged 0–4 years from most deprived areas within urban cities had higher call rates to out-of-hours GPs [34]. Hence, there is evidence that health seeking behaviors of children and their families are affected by other factors including proximity and urbanization. A 2019 report [27] concluded that residents in the Netherlands lived on average 1.0 km to the nearest GP. The same report observed that in the city of Rotterdam the proximity between residents and their GPs is on average 0.6 km. Assuming that in our study population, children from different SDA groups had equal proximity to a GP, then it would have been unlikely that our findings would be affected by proximity to GP services.

In order to assure equal access to primary healthcare services, it is important to understand the relationship between area deprivation and health seeking behaviors in the clinical context. Literature on a wide range of reasons for GP encounters amongst pediatric population is scarce [35, 36]. Of the available literature on area level social deprivation and childhood consultation rates, the majority focus primarily on one clinical diagnosis. Only a handful researched the role of socioeconomic factors and various diagnoses or symptoms according to a validated classification system [1, 25, 31]. ICPC-1 was first published in 1987 as a tool to assist GPs when they record RFEs, diagnosis/problems or processes. In 1998 the second edition (ICPC-2) was published in part due to a large contribution by the Netherlands, and has been implemented by GPs worldwide [24]. The RPCD uses a modified extended version of the ICPC-1 managed by the Dutch college of General Practitioners and not the widely used ICPC-2 system. There is considerable overlap between both systems. Furthermore, the ICPC-1 system includes additional 2-digit sub-codes expanding on more diagnoses not otherwise specified in the ICPC-2 system. For instance, symptoms related to contraception is coded as W14.00 in ICPC-2 system. In addition to W14.00 in the ICPC-1 system, the extended version includes W14.01 (pessary occlusive) and W14.02 (contraception injection). Despite the slight differences between ICPC-1 and ICPC-2 systems, our findings have important clinical implications since we included all consultations with a valid ICPC-1 code. To our knowledge this is the first study to report extensively on all ICPC-1 codes and provide accurate consultation rates per body system in a pediatric population taking into account local area deprivation level. We were able to analyse the distribution of multiple diseases across various stages in childhood development. However, contrary to our initial hypothesis, we found that adolescent females from least deprived areas had higher consultation rates within the ICPC-1 chapter pregnancy/family planning. In the Netherlands, prevention is highly emphasized in primary care with Dutch GP’s being the first point of access for contraceptives including oral, injection and placing of intrauterine devices (IUDs) [37]. This service is covered by the Dutch National health insurance and free for females under the age of 20 years. Furthermore, in the Netherlands, there are very few privately owned family planning clinics since GPs provide this care for free to the general population.

Adolescent pregnancy is regarded as a social problem worldwide not to mention the health risks associated with maternal morbidity and infant mortality [38]. Information on pregnancy and termination of pregnancy among adolescent females can be used to improve health policy and monitor progress towards reducing these rates which in turn are associated with risk of morbidity and death. Our findings are similar to existing evidence that socio-economic inequality is associated with teenage pregnancies and termination of pregnancy rates [39–41]. Between 1980 to 1990, Smith et al., [39] found a four to eight times higher rate of consultation for teenage pregnancies in the most deprived postcodes in Scotland. Our study showed a two-fold increase rate in teenage pregnancy in children living in deprived areas compared to those in non-deprived areas. We also observed a three-fold increase in induced termination of pregnancy in female adolescents from deprived areas. On the contrary, Smith [39] found that teenagers from affluent areas had a higher rate of termination of pregnancies amongst adolescent females. The differences can be in part due to the lack of individual level determinants that could explain why unwanted pregnancies or their termination occur in these groups; such as parental education level, household income, (ineffective) use of contraception, beliefs and attitudes towards unwanted pregnancy [42].

### Strengths and limitations

To our knowledge, this large population-based study to assess clinical and social trends in the use of healthcare by pediatric population in general practice setting in the Netherlands. GPs entered data during or shortly after a consultation which would increase data entry accuracy. However, one limitation is the accuracy of diagnoses in this study. For instance, we were not able to verify the diagnoses recorded by GPs, which could have led to an overestimation and underestimation of certain ICPC-1 codes. Nonetheless, the extraction method used to identify the number of consultations per year per person and the associated ICPC-1 chapter codes was accurate to assess consultation rates which is what we aimed to determine.

Consultation rates is a widely used indicator to determine workload in a general practice setting, however the complexity of consultations was not assessed in this study. For instance we did not explore various tasks performed during a consultation such as rate of prescriptions, referral rates, time spent during consultations (consultation length), investigations or managing comorbidities. Such information would provide a more accurate indication of workload which could be possible in another research using the RPCD, however is beyond the scope of this current study.

Area level social deprivation index or score as a determinant of health outcomes at the individual level is widely used and reported in existing literature. Our study lacked a cut-off point for the SDA index as well as information on individual socioeconomic status for consultation data since children were classified by an area level social deprivation score according to their area code. This may have distorted our findings due to ecological fallacy whereby the relationships we observed in both groups does not necessarily hold for the individual child. Obtaining individual-level data is complex, however, this study provides preliminary insight on the causes of morbidity in children that is driving inequalities at the local area level.

## Conclusion

Rotterdam is a metropolitan city in the Netherlands, with the largest community of ethnic minorities and the highest percentage of children who live in low-income households. We found evidence that the neighborhood in which a child lives influences consultation rate with their GP. Interestingly, we found an inverse association such that children and adolescents in the least socially deprived areas consulted their GPs more often than children and adolescents in the most socially deprived areas for pregnancy and family planning. In the most socially deprived area group, female adolescents consulted their GP less frequently for contraceptives, possibly explaining the higher rate of unwanted pregnancy and induced termination of pregnancy in this group. Both teen pregnancy and induced termination of pregnancies have been linked to detrimental social and economic impact further perpetuating the disparities in prevention and healthcare access. Our study illustrates that in order to make policy changes and allocate resources, we need to focus on the broader clinical context and intensify our research to discover the underlying health seeking behaviors at different development phases of children and adolescents. Unless appropriate measures are taken to increase and improve GP resources to reach and impact those at risk, health inequalities present at childhood will continue onwards towards adult life and elderly age.

## Supplementary Information

**Additional file 1.**

## Data Availability

The data that support the findings of this study are available from the RPCD but restrictions apply to the availability of these data, which were used under license for the current study, and so are not publicly available. Data are however available from the authors upon reasonable request and with permission of RPCD. The authors only have permission to share metadata with third parties. Metadata is referred as descriptive variable information. As study data was pseudonymised, it is not possible to obtain nor share identifiable information. Extensive measures are in place to ensure confidentiality and ethical conduct of the research. The corresponding author M. S. Fonderson can be contacted to access data.

## Abbreviations

GP: General Practitioner
ICPC: International Classification of Primary Care
ID: Identification
IPCI: Integrated Primacy Care Information
IRR: Incidence Risk Ratio
NZA: Dutch Health Authority
RFE: Reason for encounter
RPCD: Rijnmond Primary Care Database
SDA: Socially Deprived Areas
SOEP: Subjective Objective Evaluation Plan
