# Supplementary material for: The role of area level social deprivation on childhood and adolescent consultation rate in primary care: a population based, cohort study
Source: BMC Prim Care. 2022 Oct 28;23:270. doi: 10.1186/s12875-022-01873-x (PMC9613447; doi:10.1186/s12875-022-01873-x)
Supplement: Supplementary file 1 — Additional file 1. [file 12875_2022_1873_MOESM1_ESM.docx]

Supplementary


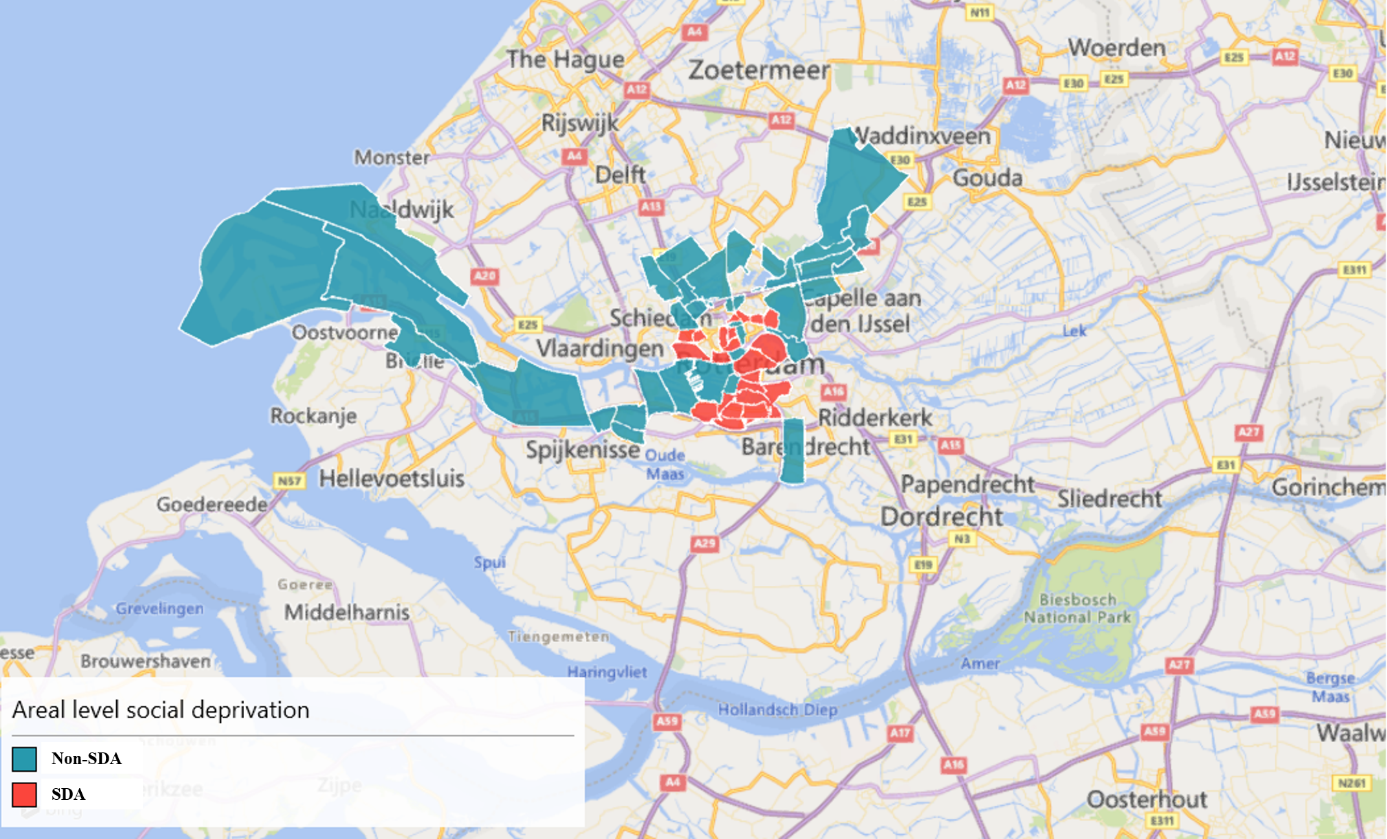


Appendix 1**.** Map showing the distribution postal area codes in 99 neighbourhoods of Rotterdam, the Netherlands, color-coded according to area level deprivation category. Information derived from the National Dutch Health Authority (NZA) report 2017. Created with Microsoft Excel ® 2016

*Appendix 2 Yearly childhood and adolescent consultation rate per 1000 person years in SDA and Non-SDA groups according to ICPC-1 chapters*

|  | **2013** | | | | **2014** | | | |  | **2015** | | |
| --- | --- | --- | --- | --- | --- | --- | --- | --- | --- | --- | --- | --- |
|  | **Incidence rate per 1000 person years** | | | | **Incidence rate per 1000 person years** | | | | **Incidence rate per 1000 person years** | | | |
| ICPC chapter | **Overall** | **SDA** | **Non-SDA** | **IRR (95% CI)** | **Overall** | **SDA** | **Non-SDA** | **IRR (95% CI)** | **Overall** | **SDA** | **Non-SDA** | **IRR (95% CI)** |
| A General Unspecified | 206 | 239 | 193 | 1.24 (1.18-1.80) | 213 | 262 | 194 | 1.35 (1.29-1.41) | 209 | 260 | 189 | 1.38 (1.32-1.44) |
| B Blood | 23 | 30 | 16 | 1.88 (1.60 -2.20) | 19 | 25 | 15 | 1.66 (1.41-1.96) | 17 | 21 | 14 | 1.57 (1.32- 1.87) |
| D Digestive | 182 | 203 | 141 | 1.44 (1.36-1.51) | 180 | 212 | 147 | 1.45 (1.38-1.52) | 179 | 210 | 148 | 1.42 (1.35-1.49) |
| F Eye | 93 | 105 | 72 | 1.47 (1.36-1.59) | 96 | 112 | 80 | 1.41 (1.31-1.51) | 97 | 124 | 77 | 1.62 (1.52-1.74) |
| H Ear | 132 | 115 | 115 | 1.00 (0.93-1.07) | 126 | 117 | 116 | 1.01 (0.94-1.08) | 135 | 127 | 123 | 1.03 (0.97-1.10) |
| K Cardiovascular | 14 | 14 | 11 | 1.29 (1.04-1.59) | 13 | 13 | 11 | 1.14 (0.92-1.42) | 12 | 15 | 10 | 1.56 (1.27-1.92) |
| L Musculoskeletal | 179 | 164 | 154 | 1.06 (1.00 -1.12) | 188 | 177 | 174 | 1.02 (0.97-1.07) | 184 | 174 | 177 | 0.99 (0.94-1.04) |
| N Neurological | 64 | 67 | 52 | 1.28 (1.16-1.41) | 65 | 70 | 57 | 1.24 (1.14-1.36) | 64 | 69 | 56 | 1.25 (1.14-1.37) |
| P Psychological | 97 | 82 | 86 | 0.95 (0.88-1.03) | 111 | 101 | 103 | 0.98 (0.91-1.05) | 115 | 104 | 107 | 0.97 (0.90-1.04) |
| R Respiratory | 316 | 337 | 251 | 1.35 (1.30 -1.40) | 305 | 332 | 260 | 1.28 (1.23-1.32) | 314 | 344 | 270 | 1.28 (1.23-1.32) |
| S Skin | 372 | 364 | 309 | 1.18 (1.14-1.22) | 376 | 378 | 335 | 1.13 (1.09-1.16) | 363 | 367 | 324 | 1.13 (1.10-1.17) |
| T Endocrine | 51 | 69 | 35 | 1.99 (1.79-2.20) | 45 | 64 | 32 | 2.00 (1.80-2.22) | 46 | 66 | 34 | 1.95 (1.76-2.16) |
| U Urological | 41 | 38 | 34 | 1.11 (0.97-1.25) | 39 | 37 | 36 | 1.03 (0.91-1.16) | 41 | 41 | 37 | 1.13 (1.00-1.27) |
| W Pregnancy | 24 | 13 | 23 | 0.59 (0.59-0.72) | 23 | 13 | 25 | 0.53 (0.43-0.64) | 24 | 16 | 24 | 0.64 (0.54-0.77) |
| X Female Genitalia | 29 | 26 | 25 | 1.04 (0.89-1.21) | 29 | 27 | 26 | 1.05 (0.91-1.22) | 30 | 30 | 27 | 1.13 (0.98-1.30) |
| Y Male Genitalia | 26 | 26 | 21 | 1.22 (1.05-1.43) | 27 | 27 | 24 | 1.15 (0.99-1.33) | 25 | 26 | 22 | 1.16 (1.00-1.35) |
| Z Social | 22 | 33 | 13 | 2.61 (2.23-3.07) | 18 | 23 | 14 | 1.70 (1.43-2.02) | 15 | 18 | 12 | 1.44 (1.19-1.74) |

| *Appendix 2 (cont.)* |  | **2016** | | |  | **2017** | | | **2018** | | | |
| --- | --- | --- | --- | --- | --- | --- | --- | --- | --- | --- | --- | --- |
|  | **Incidence rate per 1000 person years** | | | | **Incidence rate per 1000 person years** | | | | **Incidence rate per 1000 person years** | | | |
| ICPC-1 chapter | **Overall** | **SDA** | **Non-SDA** | **IRR (95% CI)** | **Overall** | **SDA** | **Non-SDA** | **IRR (95% CI)** | **Overall** | **SDA** | **Non-SDA** | **IRR (95% CI)** |
| A General Unspecified | 219 | 275 | 198 | 1.39 (1.33-1.44) | 204 | 264 | 185 | 1.42 (1.37-1.48) | 221 | 258 | 210 | 1.23 (1.18-1.28) |
| B Blood | 18 | 21 | 15 | 1.42 (1.21-1.67) | 18 | 21 | 14 | 1.57 (1.34-1.84) | 16 | 18 | 14 | 1.28 (1.08-1.51) |
| D Digestive | 179 | 205 | 148 | 1.38 (1.32-1.45) | 172 | 184 | 141 | 1.30 (1.24-1.37) | 176 | 190 | 152 | 1.25 (1.19-1.31) |
| F Eye | 93 | 117 | 74 | 1.58 (1.47-1.68) | 93 | 113 | 72 | 1.58 (1.48-1.68) | 87 | 100 | 74 | 1.35 (1.26-1.45) |
| H Ear | 130 | 121 | 119 | 1.02 (0.96-1.08) | 127 | 109 | 113 | 0.97 (0.91-1.03) | 124 | 107 | 116 | 0.92 (0.86-0.98) |
| K Cardiovascular | 12 | 13 | 10 | 1.21 (0.98-1.48) | 13 | 12 | 11 | 1.12 (0.91-1.03) | 12 | 12 | 11 | 1.14 (0.93-1.39) |
| L Musculoskeletal | 185 | 174 | 169 | 1.03 (0.98-1.08) | 185 | 163 | 165 | 0.99 (0.94-1.04) | 182 | 157 | 170 | 0.92 (0.87-0.97) |
| N Neurological | 60 | 69 | 50 | 1.37 (1.26-1.50) | 62 | 66 | 51 | 1.29 (1.18-1.40) | 61 | 65 | 53 | 1.23 (1.13-1.34) |
| P Psychological | 114 | 107 | 104 | 1.03 (0.96-1.10) | 115 | 100 | 103 | 0.98 (0.92-1.04) | 113 | 100 | 105 | 0.95 (0.89-1.01) |
| R Respiratory | 321 | 351 | 274 | 1.28 (1.24-1.32) | 293 | 308 | 243 | 1.27 (1.23--1.31) | 282 | 293 | 247 | 1.18 (1.14-1.23) |
| S Skin | 356 | 357 | 316 | 1.13 (1.10-1.17) | 353 | 347 | 300 | 1.16 (1.12-1.19) | 352 | 327 | 320 | 1.02 (0.99-1.05) |
| T Endocrine | 42 | 60 | 31 | 1.91 (1.73-2.11) | 42 | 57 | 31 | 1.83 (1.66-2.02) | 43 | 59 | 33 | 1.76 (1.60-1.93) |
| U Urological | 40 | 38 | 36 | 1.06 (0.94-1.19) | 36 | 34 | 32 | 1.08 (0.96-1.21) | 37 | 35 | 33 | 1.05 (0.94-1.18) |
| W Pregnancy | 23 | 13 | 24 | 0.53 (0.44-0.64) | 24 | 11 | 24 | 0.48 (0.39-0.57) | 26 | 11 | 27 | 0.38 (0.32-0.47) |
| X Female Genitalia | 28 | 27 | 25 | 1.10 (0.96-1.26) | 28 | 25 | 25 | 1.00 (0.88-1.15) | 28 | 23 | 26 | 0.88 (0.77-1.01) |
| Y Male Genitalia | 24 | 27 | 21 | 1.30 (1.13-1.50) | 25 | 25 | 20 | 1.24 (1.08-1.42) | 24 | 22 | 22 | 1.02 (0.88-1.18) |
| Z Social | 15 | 21 | 12 | 1.76 (1.48-2.08) | 16 | 19 | 12 | 1.59 (1.35-1.88) | 14 | 17 | 12 | 1.49 (1.25-1.77) |

| *Appendix 2 (cont.)* | **2019** | | | |
| --- | --- | --- | --- | --- |
|  | **Incidence rate per 1000 person years** | | | |
| ICPC-1 chapter | **Overall** | **SDA** | **Non-SDA** | **IRR (95% CI)** |
| A General Unspecified | 230 | 286 | 213 | 1.34 (1.30 - 1.39) |
| B Blood | 16 | 19 | 13 | 1.48 (1.26 - 1.75) |
| D Digestive | 174 | 187 | 144 | 1.30 (1.24 - 1.36) |
| F Eye | 92 | 106 | 74 | 1.48 (1.34 - 1.53) |
| H Ear | 127 | 109 | 113 | 0.96 (0.91 - 1.03) |
| K Cardiovascular | 11 | 12 | 9 | 1.25 (1.02 -1.54) |
| L Musculoskeletal | 181 | 160 | 160 | 1.00 (0.95 - 1.05) |
| N Neurological | 61 | 67 | 50 | 1.34 (1.23 - 1.45) |
| P Psychological | 118 | 106 | 105 | 1.02 (0.95 - 1.08) |
| R Respiratory | 291 | 301 | 245 | 1.23 (1.19 - 1.27) |
| S Skin | 350 | 329 | 304 | 1.08 (1.05 - 1.12) |
| T Endocrine | 43 | 59 | 32 | 1.82 (1.65 - 2.00) |
| U Urological | 37 | 36 | 33 | 1.10 (0.98 - 1.23) |
| **W Pregnancy** | **27** | **13** | **28** | **0.46 (0.38 - 0.55)** |
| X Female Genitalia | 28 | 27 | 24 | 1.11 (0.98 - 1.27) |
| Y Male Genitalia | 25 | 24 | 21 | 1.13 (0.98 - 1.30) |
| Z Social | 15 | 17 | 12 | 1.38 (1.16 - 1.65) |

| *Appendix 3* Pregnancy & Family Planning *consultation rate per 1000 person years.  Incidence Risk Ratio (IRR) and 95% confidence interval (CI)* | | | | | |
| --- | --- | --- | --- | --- | --- |
|  |  | **0verall** | **SDA** | **Non-SDA** | **SDA vs Non-SDA** |
| **ICPC-1 chapter code** | **ICPC-1 chapter description** | **N (IR per 1000 person years)** | **N (IR per 1000 person years)** | **N (IR per 1000 person years)** | **IRR (95% CI)** |
| W01.00 | Question of pregnancy | 15 (0.06) | 6 (0.09) | 9 (0.04) | 1.96 (0.70-5.50) |
| W02.00 | Fear of pregnancy | 100 (0.41) | 36 (0.52) | 64 (0.31) | 1.65 (1.10-2.48) |
| W03 cluster | Antepartum bleeding | 5 (0.02) | 1 (0.01) | 4 (0.02) | 0.73 (0.08-6.57) |
| W05.00 | Pregnancy vomiting/nausea | 9 (0.03) | 2 (0.03) | 7 (0.03) | 0.84 (0.17-4.04) |
| W10 cluster | Contraception postcoital | 16 (0.07) | 2 (0.03) | 14 (0.07) | 0.42 (0.10-1.85) |
| *W10.01* | *Contraception postcoital* | *5 (0.02)* | *1 (0.01)* | *4 (0.02)* | *0.73 (0.08-6.57)* |
| *W10.02* | *Morning after pill* | *11 (0.04)* | *1 (0.01)* | *10 (0.05)* | *0.29 (0.04-2.29)* |
| W11.00 | Contraception oral | 3588 (14.5) | 369 (5.71) | 3192 (15.7) | 0.36 (0.33-0.40)* |
| W12.00 | Contraception intrauterine | 217 (0.88) | 21 (0.30) | 196 (0.96) | 0.31 (0.20-0.49)* |
| W13.00 | Sterilization female | 0 | 0 | 0 | 0 |
| *W14 cluster* | *Contraception other* | *803 (3.23)* | *183 (2.64)* | *620 (3.0)* | *0.87 (0.74-1.02)* |
| W14.00 | Contraception other | 596 (2.42) | 151 (2.18) | 445 (2.19) | 1.00 (0.83-1.20) |
| W14.01 | Pessary occlusive | 0 | 0 | 0 | 0 |
| W14.02 | Contraception injection | 248 (1.08) | 41 (0.59) | 207 (1.02) | 0.58 (0.42-0.81)* |
| W15.00 | Infertility /subfertility | 3 (0.012) | 2 (0.028) | 1 (0.005) | 5.87 (0.53-64.77 |
| W17.00 | Postpartum bleeding | 0 | 0 | 0 | 0 |
| W18.00 | Postpartum symptom/complaint other | 4 (0.02) | 2 (0.03) | 2 (0.01) | 2.94 (0.41-20.85) |
| W19.00 | Lactation symptom/complaint | 31 (0.13) | 10 (0.14) | 21 (0.10) | 1.40 (0.66-2.97) |
| W20.00 | Other symptom/complaint breast/pregnancy/childbirth | 14 (0.06) | 4 (0.06) | 11 (0.05) | 1.17 (0.37-3.75) |
|  |  |  |  |  |  |

| *Appendix 3 (cont.*) | | | | | |
| --- | --- | --- | --- | --- | --- |
|  |  | **0verall** | **SDA** | **Non-SDA** | **SDA vs Non-SDA** |
| **ICPC-1 chapter code** | **ICPC-1 chapter description** | **N (IR per 1000 person years)** | **N (IR per 1000 person years)** | **N (IR per 1000 person years)** | **IRR (95% CI)** |
| W27.00 | Fear of complications of pregnancy | 3 (0.01) | 1 (0.01) | 2 (0.01) | 1.47 (0.14-16.2) |
| W28.00 | Limited function/disability (W) | 1 (0.004) | 0 | 1 (0.005) | 0 (0.00-NaN) |
| W29.00 | Pregnancy symptom/complaint other | 8 (0.03) | 1 (0.01) | 7 (0.03) | 0.42 (0.05-3.41) |
| *W30.00 -W49.00 cluster* | *Diagnostic, screening and prevention* | *1 (0.004)* | *0* | *1 (0.005)* | *0 (0.00-NaN)* |
| *W50.00 -W59.00 cluster* | *Medication, treatment, procedure* | *0* | *0* | *0* | *0* |
| *W60.00 -W61.00 cluster* | *Test results* | *2 (0.008)* | *0* | *2 (0.01)* | *0 (0.00-NaN)* |
| *W62.00 cluster* | *Administrative* | *1 (0.004)* | *0* | *1 (0,005)* | *0 (0.00-NaN)* |
| *W63.00-W69.00 cluster* | *Referrals* | *0* | *0* | *0* | *0* |
| *W70 cluster* | *Puerperal infection/sepsis* | *4 (0.02)* | *1 (0.01)* | *3 (0.02)* | *0.98 (0.10-9.41)* |
| W71.00 | Other infection complicating pregnancy/puerperium | 8 (0.03) | 2 (0.03) | 6 (0.03) | 0.98 (0.40-4.85) |
| W72.00 | Malignant neoplasm related to pregnancy | 0 | 0 | 0 | 0 |
| W73.00 | Benign/unspecified neoplasm related to pregnancy | 0 | 0 | 0 | 0 |
| W75.00 | Injury complicating pregnancy | 0 | 0 | 0 | 0 |
| W76.00 | Congenital anomaly complicating pregnancy | 9 (0.04) | 4 (0.06) | 5 (0.03) | 2.35 (0.63-8.75) |
| *W77 cluster* | *Varices/Hemorrhoids/DVT during pregnancy* | *2 (0.01)* | *2 (0.03)* | *0* | *Inf (Nan-Inf)* |
| W78.00 | Pregnancy | 59 (0.25) | 25 (0.36) | 34 (0.17) | 2.16 (1.29-3.62)* |
| W79.00 | Unwanted pregnancy | 76 (0.31) | 32 (0.46) | 44 (0.44) | 2.14 (1.35-3.37)* |
| W80.00 | Ectopic pregnancy | 0 | 0 | 0 | 0 |
|  |  |  |  |  |  |

| *Appendix 3 (cont.)* | | | | | |
| --- | --- | --- | --- | --- | --- |
|  |  | **0verall** | **SDA** | **Non-SDA** | **SDA vs Non-SDA** |
| **ICPC-1 chapter code** | **ICPC-1 chapter description** | **N (IR per 1000 person years)** | **N (IR per 1000 person years)** | **N (IR per 1000 person years)** | **IRR (95% CI)** |
| W81.00 | Toxemia of pregnancy | 0 | 0 | 0 | 0 |
| W82.00 | Spontaneous abortion | 11 (0.04) | 5 (0.07) | 6 (0.03) | 2.45 (0.75-8.02) |
| W83.00 | Induced abortion | 40 (0.16) | 20 (0.29) | 20 (0.10) | 2.94 (1.58-5.46) |
| *W84 cluster* | *Pregnancy high risk* | *34 (0.14)* | *6 (0.09)* | *28 (0.14)* | *0.63 (0.26-1.52)* |
| W84.00 | Pregnancy high risk | 11 (0.05) | 0 | 11 (0.05) | 0 (0.00-NaN) |
| W84.01 | Urological infection during pregnancy | 0 | 0 | 0 | 0 |
| W84.02 | Gestational diabetes | 2 (0.01) | 1 (0.01) | 1 (0.01) | 2.94 (0.18-46.95) |
| W84.04 | Growth retardation/abnormal fetal growth | 6 (0.02) | 1 (0.014) | 5  (0.03) | 0.59 (0.07-5.03) |
| W84.05 | Disproportion | 4 (0.02) | 0 | 4 (0.02) | 0 (0-NaN) |
| W84.06 | Cervical incompetence | 0 | 0 | 0 | 0 |
| W84.07 | Location fetus deviation | 8 (0.04) | 2 (0.03) | 6 (0.03) | 0.98 (0.20-4.85) |
| *W80.08* | *Blood group /rhesus antagonism* | *2 (0.01)* | *1 (0.01)* | *1 (0.01)* | *2.94 (0.18-46.95)* |
| W90.00 | Uncomplicated labor/delivery live birth | 1080 (4.38) | 160 (2.31) | 920 (4.52) | 0.51 (0.43-0.60)* |
| W91.00 | Uncomplicated labor/delivery stillbirth | 2 (0.01) | 0 | 2 (0.01) | 0 (0.00-NaN) |
| *W92 cluster* | *Complicated labor/delivery live birth* | *279 (1.13)* | *71 (1.02)* | *208 (1.02)* | *1.00 (0.77-1.31)* |
| W92.00 | Complicated labor delivery/live birth | 76 (0.31) | 28 (0.40) | 48 (0.24) | 1.71 (1.07-2.73) |
| W92.01 | Cesarean section | 163 (0.66) | 40 *0.58) | 123 (0.60) | 0.95 (0.67-1.36) |
| W92.02 | Vacuum extraction | 41 (0.16) | 3 (0.04) | 38 (0.19) | 0.23 (0.07-0.75) |
| W92.03 | Forceps extraction | 0 | 0 | 0 | 0 |
| W93.00 | Complicated labor/delivery stillbirth | 5 (0.02) | 1 (0.01) | 4 (0.02) | 0.73 (0.08-6.57) |
|  |  |  |  |  |  |
| *Appendix 3 (cont.*) | | | | | |
|  |  | **0verall** | **SDA** | **Non-SDA** | **SDA vs Non-SDA** |
| **ICPC-1 chapter code** | **ICPC-1 chapter description** | **N (IR per 1000 person years)** | **N (IR per 1000 person years)** | **N (IR per 1000 person years)** | **IRR (95% CI)** |
| W94.00 | Puerperal mastitis | 21 (0.09) | 1 (0.01) | 20 (0.10) | 0.23 (0.07-0.75) |
| W95.00 | Breast disorder in pregnancy other | 3 (0.02) | 3 (0.04) | 0 | Inf (Nan-Inf) |
| W96.00 | Complications of puerperium other | 10 (0.04) | 6 (0.09) | 4 (0.02) | 4.40 (1.24-15.61) |
| W99.00 | Disorder of pregnancy/delivery other | 8 (0.03) | 5 (0.07) | 3 (0.02) | 4.89 (1.17-20.48) |

|  |
| --- |
